# Supplementary material for: Electrospun SnO2/WO3 Heterostructure Nanocomposite Fiber for Enhanced Acetone Vapor Detection
Source: Polymers (Basel). 2023 Nov 3;15(21):4318. doi: 10.3390/polym15214318 (PMC10647394; doi:10.3390/polym15214318)
Supplement: Supplementary file 1 [file polymers-15-04318-s001.zip › polymers-2668172-supplementary.pdf]

Supplementary Materials

Electrospun SnO_2/WO_3 Heterostructure Nanocomposite Fiber for Enhanced Acetone Vapor Detection

Ting-Han Lin [1†], Yin-Hsuan Chang [1†], Ting-Hung Hsieh [1†], Yu-Ching Huang [1,2*], and Ming-Chung Wu [1,2,3,4*]

[1] Department of Chemical and Materials Engineering, Chang Gung University, Taoyuan 33302, Taiwan
[2] Department of Materials Engineering, Ming-Chi University of Technology, New Taipei City 24301, Taiwan
[3] Center for Sustainability and Energy Technologies, Chang Gung University, Taoyuan 33302, Taiwan
[4] Division of Neonatology, Department of Pediatrics, Chang Gung Memorial Hospital at Linkou, Taoyuan 33305, Taiwan

* Correspondence: Y.-C. Huang: huangyc@mail.mcut.edu.tw; M.-C. Wu: mingchungwu@cgu.edu.tw
† These authors contributed equally to this work as first author.

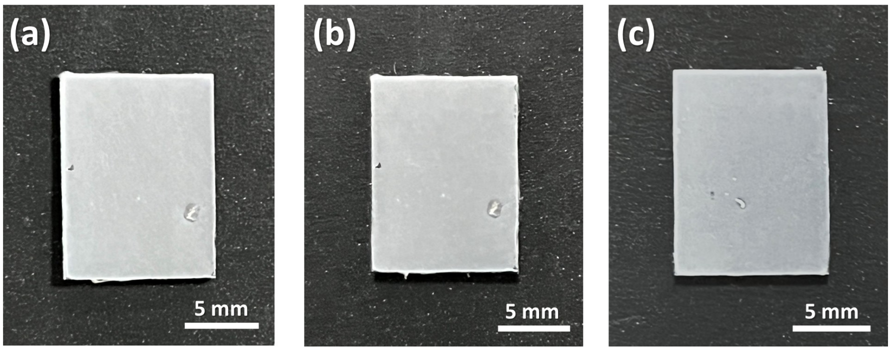

Figure S1. Photographs of the PMMA-Ag-SnO_2/WO_3 sensing fibers: (a) in initial state, (b) post UV-Ozone treatment, and (c) exposure to acetone vapor.

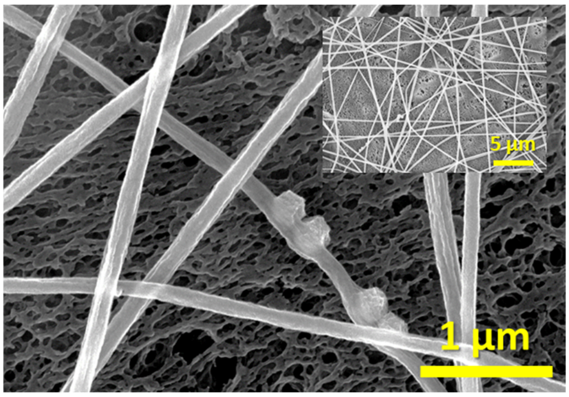

Figure S2. Morphology of PMMA-Ag-SnO_2/WO_3 fibers with UV ozone treatment.

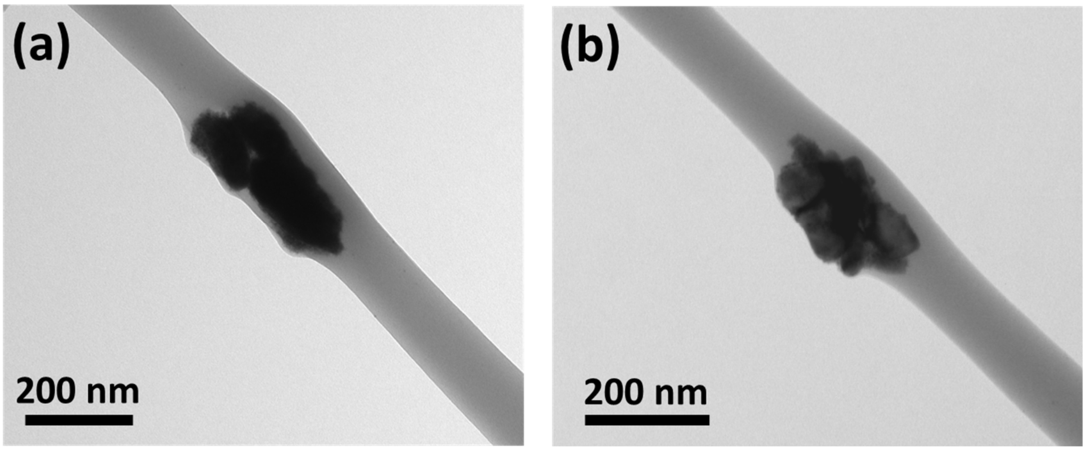

Figure S3. TEM images of PMMA-Ag-SnO_2/WO_3 fibers (a) without and (b) with UV-ozone treatment.

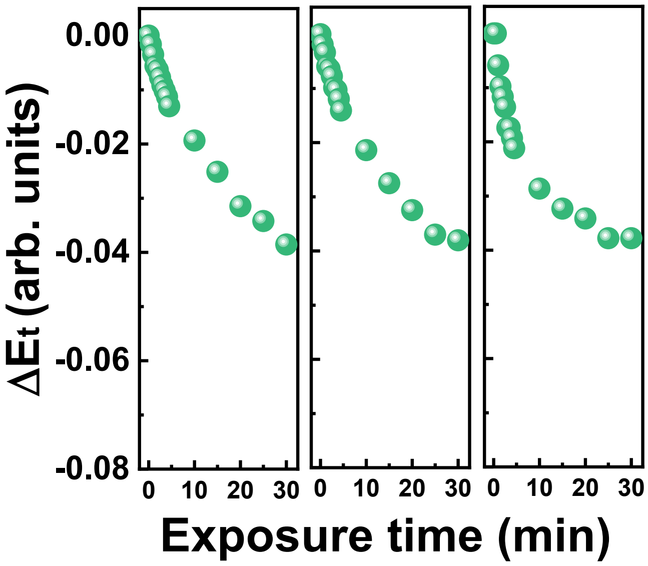

Figure S4. Reproducibility test of PMMA-Ag-SnO_2/WO_3 fibers exposed to 100 ppm acetone vapor.

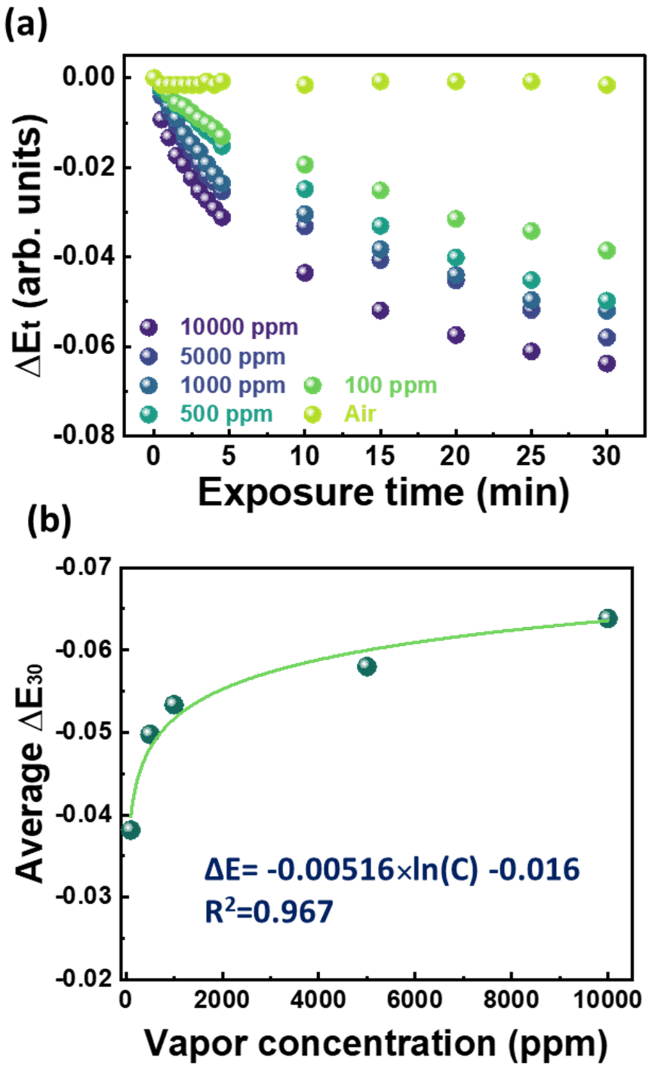

Figure S5. (a) Extinction change of PMMA-Ag-SnO_2/WO_3 fibers exposed to acetone with various concentration ppm over exposure time, t min and (b) the calibration curve corresponding to 30-min exposure of PMMA-Ag-SnO_2/WO_3 fibers to acetone vapor.

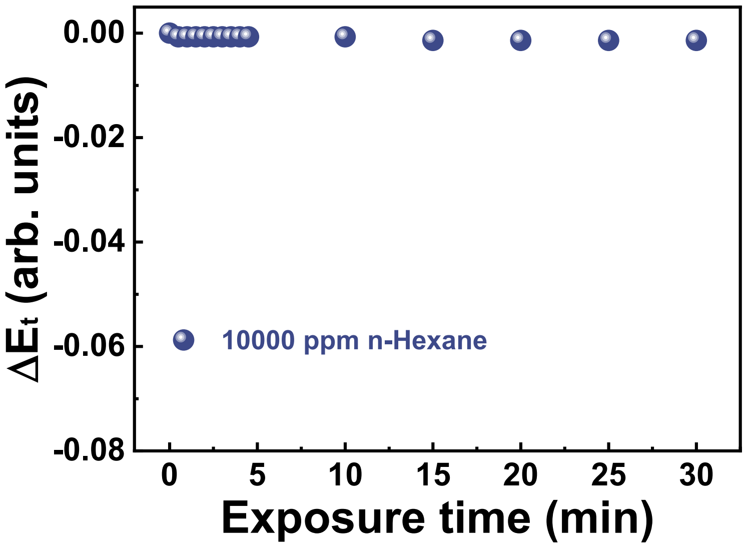

Figure S6. Extinction change of PMMA-Ag-SnO_2/WO_3 fibers exposed to 10,000 ppm n-hexane over exposure time, t min.
